# A Systematic Review of Cardio-Metabolic Properties of *Lonicera caerulea* L.

**DOI:** 10.3390/antiox13060694

**Published:** 2024-06-05

**Authors:** Larisa Bora, Adelina Lombrea, Stefan Laurentiu Batrina, Valentina Oana Buda, Oana-Maria Esanu, Oana Pasca, Cristina Adriana Dehelean, Stefania Dinu, Zorita Diaconeasa, Corina Danciu

**Affiliations:** 1 Department of Pharmacognosy, Faculty of Pharmacy, “Victor Babeș” University of Medicine and Pharmacy, Eftimie Murgu Square, No. 2, 300041 Timisoara, Romania; larisa.bora@umft.ro (L.B.); adelina.lombrea@umft.ro (A.L.); corina.danciu@umft.ro (C.D.); 2 Research and Processing Center for Medicinal and Aromatic Plants, “Victor Babeș” University of Medicine and Pharmacy, Eftimie Murgu Square, No. 2, 300041 Timisoara, Romania; cadehelean@umft.ro; 3 Department of Crop Science, Faculty of Agriculture, University of Life Sciences “King Mihai I” from Timisoara, Calea Aradului 119, 300645 Timisoara, Romania; 4 Discipline of Clinical Pharmacy, Communication in Pharmacy, Pharmaceutical Care, Faculty of Pharmacy, “Victor Babeș” University of Medicine and Pharmacy, Eftimie Murgu Square, No. 2, 300041 Timisoara, Romania; 5 Research Center for Pharmaco-Toxicological Evaluation, “Victor Babeș” University of Medicine and Pharmacy, Eftimie Murgu Square, No. 2, 300041 Timisoara, Romania; 6 Faculty of Pharmacy, “Victor Babeș” University of Medicine and Pharmacy, Eftimie Murgu Square, No. 2, 300041 Timisoara, Romania; oana-maria.esanu@student.umft.ro (O.-M.E.); oana.pasca@student.umft.ro (O.P.); 7 Department of Toxicology and Drug Industry, Faculty of Pharmacy, “Victor Babeș” University of Medicine and Pharmacy, Eftimie Murgu Square, No. 2, 300041 Timisoara, Romania; 8 Department of Pedodontics, Faculty of Dental Medicine, “Victor Babes” University of Medicine and Pharmacy Timisoara, 9 No., Revolutiei Bv., 300041 Timisoara, Romania; dinu.stefania@umft.ro; 9 Pediatric Dentistry Research Center, Faculty of Dental Medicine, “Victor Babes” University of Medicine and Pharmacy Timisoara, 9 No., Revolutiei Bv., 300041 Timisoara, Romania; 10 Department of Food Science and Technology, Faculty of Food Science and Technology, University of Agricultural Science and Veterinary Medicine, Calea Manastur, 3-5, 400372 Cluj-Napoca, Romania; zorita.sconta@usamvcluj.ro

**Keywords:** *Lonicera caerulea* L., cardiovascular diseases, metabolic syndrome, antioxidant, hypolipidemic, hypoglycemic, hepatoprotective, vasoprotective

## Abstract

In the light of growing concerns faced by Western societies due to aging, natality decline, and epidemic of cardio-metabolic diseases, both preventable and treatable, new and effective strategical interventions are urgently needed in order to decrease their socio-economical encumbrance. The recent focus of research has been redirected towards investigating the potential of haskap (*Lonicera caerulea* L.) as a novel functional food or superfruit. Therefore, our present review aims to highlight the latest scientific proofs regarding the potential of *Lonicera caerulea* L. (LC), a perennial fruit-bearing plant rich in polyphenols, in reversing cardio-metabolic dysfunctions. In this regard, a systematic search on two databases (PubMed and Google Scholar) from 1 January 2016 to 1 December 2023 was performed, the keyword combination being *Lonicera caerulea* L. AND the searched pharmacological action, with the inclusion criteria consisting of in extenso original articles, written in English. The health-enhancing characteristics of haskap berries have been examined through in vitro and in vivo studies from the 35 included original papers. Positive effects regarding cardiovascular diseases and metabolic syndrome have been assigned to the antioxidant activity, hypolipidemic and hypoglycemic effects, as well as to the hepatoprotective and vasoprotective potential. Latest advances regarding LCF mechanisms of action are detailed within this review as well. All these cutting-edge data suggest that this vegetal product would be a good candidate for further clinical studies.

## 1. Introduction

### 1.1. Evolution of Phytotherapy in the 21st Century

Phytotherapy continues to play a significant role in the 21st century as a domain for research and clinical applications. In an era of technological and scientific advancements, phytotherapy has adapted to incorporate new knowledge and approaches, contributing to its ongoing evolution and integration into modern medicine [1,2].

Recent research has underscored the importance of phytotherapy in personalized medicine, highlighting its potential in individualized healthcare [3,4]. In this regard, standardization of plant extracts, the identification and characterization of bioactive compounds, and understanding the molecular mechanisms of interaction between medicinal plants and the human body have become crucial research objectives [5].

Rigorous analysis of clinical study results brings significant validation to the efficacy of phytotherapy, solidifying its status in the modern therapeutic arsenal. Evidence-based approaches and robust research methodologies have shaped a solid scientific framework for actual phytotherapeutic practice [6]. In a world dominated by synthetic pharmaceuticals, the renewed interest in plant-based medicine reflects a desire for remedies closely connected to nature [7].

The 21st century has seen a revival in the understanding of plant compounds, driven by advancements in analytical technologies and a renewed interest in traditional botanical knowledge. This resurgence is not simply a return to past practices, but a step forward that combines ancient wisdom with modern scientific rigor [8].

*Lonicera caerulea* L. (Siberian blueberry), known for its extensive use in traditional medicine, exemplifies this evolution. By examining its potential therapeutic advantages, it can be observed how it embodies the fusion of historical effectiveness and contemporary scientific scrutiny that defines phytotherapy [9].

### 1.2. Taxonomy

*Lonicera caerulea* L., recognized by various appellations such as blue honeysuckle, sweet berry honeysuckle, fly honeysuckle, blue-berried honeysuckle, or the honeyberry, manifests as a deciduous shrub that eschews climbing tendencies. Its native habitat spans the cool-temperature regions of North America, Europe, and Asia. Furthermore, linguistically inspired by the indigenous Ainu people of Japan, the plant was named the “elixir of life” [10,11].

LC is a plant classified toxicologically in the Kingdom *Plantae*. In the taxonomic hierarchy, it belongs to the subkingdom *Tracheobionta*, and is part of the superdivision *Spermatophyta*, being included in the division *Magnoliophyta*. The specific class for this plant is *Magnoliopsida*. An additional classification places it in the subclass *Asteridae*, and from the point of view of the order, it is classified in the *Dipsacales*. This plant species belongs to the family *Caprifoliaceae*. The genus of this plant is *Lonicera*, and its distinctive species is *Lonicera caerulea* L. [12].

One widely recognized classification scheme identifies nine botanical varieties of LC, each associated with specific geographical regions. *Lonicera caerulea var. altaica*—this variety is primarily found in Northern Asia, it thrives in cold climates and is known for its adaptability to harsh weather conditions [13]. *Lonicera caerulea var. caerulea*, indigenous to Eastern Asia, is well-adapted to temperate climates and its common name is honeyberry [14]. *Lonicera caerulea var. cauriana* is native to Western North America and is well-suited to the cool and moist conditions of the Montana Northwest [15]. *Lonicera caerulea var. dependens* occurs naturally in Central Asia, including countries like Kazakhstan and Uzbekistan, and shows vigorous growth and high fruit production, adapted to the continental climate of the region [16]. *Lonicera caerulea var. edulis* (synonym: *L. edulis)* is highly valued for its edible berries and it originates from Eastern Asia [17]. *Lonicera caerulea var. emphyllocalyx* is commonly known as haskap [18]. *Lonicera caerulea var. kamtschatica*, found in Siberia, is well-adapted to the cold and harsh conditions of the area. It is known for its small but flavorful berries and is often enjoyed fresh or used in preserves [19]. *Lonicera caerulea var. pallasii*, distributed in Northern Asia and northeastern Europe, can be found in countries like Russia, Tuva, and Estonia [20]. *Lonicera caerulea var. villosa*, native to Eastern North America, specifically in regions like Vermont, Rhode Island, is commonly known as the mountain fly honeysuckle. It is well-adapted to the cool and humid conditions of the region and is often found growing in mountainous areas [21].

### 1.3. Botanical Aspects and Morphoanatomical Characteristics

These leaves have a simple morphology, meaning they are undivided and have smooth edges, giving them an overall seamless appearance. In terms of shape, the leaves are generally oval or oblong–oval, with some variation in size depending on the stage of plant development. When it comes to color, Siberian blueberry leaves are usually shades of dark blue. The flowers are arranged in inflorescences, which are clusters or groups of flowers that are held together on a common stalk. These inflorescences contribute to a visually striking display when the plant is in bloom. The flowers themselves are characterized by a predominantly white or slightly yellowish hue, adding a touch of elegance to their appearance [9,22]. In terms of morphology, these flowers have a tubular shape. This tubular structure gives them a unique and distinctive aesthetic, setting them apart from other flowers [23].

One of the distinguishing features of this plant is the production of small and round fruits. These fruits, considered berries, mature to a dark blue to violet or black color. Beyond their aesthetic appeal, these berries are rich in antioxidants, vitamins, and phytochemical compounds, contributing to heightened scientific interest in their applications in healthy nutrition and medical contexts [24].

### 1.4. Chemical Composition

According to the literature, *Lonicera caerulea* L. fruits (LCFs) are rich in phenolic compounds, such as anthocyanins, flavonoids, proanthocyanidins, and phenolic acids [25]. It is well known that the type and amount of phenolic compounds, as well as other chemical compounds, can be affected by climatic conditions, plant maturity, genetic diversity, extraction methods, and even storage conditions [26,27]. In addition to these factors, the altitude and the solar radiation can significantly influence the chemical composition [28,29].

The composition of LCFs predominantly contains polyphenolic compounds, with a significant percentage up to 79–92% of the total anthocyanin content and more than 60% of the total phenolic content being represented by cyanidin 3-O-glucoside (C3G) [30]. Gorzelany et al. established that the phenolic content of LC varies depending on the cultivar. They observed that the anthocyanin content represented 94% of the total polyphenol content, with C3G being the most important anthocyanin (approximately 82.2%). Moreover, the researchers showed that the ”Duet” cultivar of *Lonicera caerulea* var. *kamtschatica* (cultivated from Tyczyn, Poland) had the highest level of C3G (382.18 mg/100 g dry weight (DW)), while the ”Colin” cultivar of *Lonicera caerulea* var. *emphyllocalyx* (collected from Grabownica Starzeńska, Poland) had the lowest level of C3G (259.41 mg/100 g DW) among the studied cultivars [31]. Auzanneau et al. also confirmed that C3G was the main phenolic compound identified in seven LCF cultivars (ethanolic extracts, acidified with 1% formic acid) tested over three harvesting years in Valais, Switzerland. Their C3G concentrations ranged between 12.5 mg/g DW and 87.5 mg/g DW. Chlorogenic acid was the second most concentrated phenolic compound identified in haskap berries (2.29–10.1 mg/g DW) [32]. Following the evaluation of 80% methanolic extracts of twenty LCF cultivars (harvested in Heilongjiang, China), Zhang et al. established that C3G is also the major phytochemical compound found among the phenolics (911.28–10,076.58 mg/100 g DW) [33].

The type of extraction, namely the extract which was obtained by homogenization, as well as the extraction duration, are crucial factors for obtaining clear and effective results [34,35]. In this regard, Senica et al. validated that the chemical composition of LC species can be influenced by the type and duration of extraction. The ”Aurora” cultivar of LC was harvested from Šmartno pri Litiji, Slovenia. The highest phenolic content was found in the spread (1753.54 mg/100 g DW) and smoothie (1108.25 mg/100 g DW) products, while the lowest values were found in the juice (373.45 mg/100 g) and infusion (196.61 mg/100 g DW) products. High chlorogenic acid levels were observed in the spread (342.10 mg/100 g DW) and smoothie (192.49 mg/100 g DW) products of LC cultivar ”Aurora”. In the case of infusion, the soaking may occur too quickly for the active principles to be fully extracted [36].

Raudone et al. investigated eight varieties of the LC species, originating from different countries in order to demonstrate that their composition is variable depending on the origin and genotype. The cultivars ”Amphora”, ”Indigo Gem”, and ”Tundra” originate from Canada. The cultivars ”Wojtek”, ”Iga”, and ”Tola” originate from Poland, while the cultivars ”Leningradskij Velikan” and ”Nimfa” originate from Russia. The identification of phenolic acids, proanthocyanidins, and flavonoids of 80% ethanolic LCF extracts (the fruits were cultivated in Lithuania) was carried out using high-performance liquid chromatography (HPLC)-PDA and UPLC-PDA. The ”Amphora” cultivar was demonstrated to have the highest anthocyanin content (approximately 48 mg/g). This cultivar was followed by ”Indigo Gem”, ”Nimfa”, ”Tundra”, and ”Leningradskij Velikan”, with anthocyanin contents ranging between 19 and 31 mg/g, while the lowest anthocyanin content was represented by the ”Tola”, ”Wojtek”, and ”Iga” cultivars (0.46, 1.63 and 5.39 mg/g, respectively) [37]. Similar to the abovementioned research of Gorzelany et al. [31], this study demonstrated that the most abundant anthocyanin among the eight varieties was C3G, which accounts for between 84% and 89% of the total anthocyanin [37]. The Russian cultivar ”Leningradskij Velikan” and the Canadian cultivars ”Amphora” and ”Indigo Gem” showed a high total content of flavonoids. Rutin was found in the highest concentration in the ”Indigo Gem” cultivar from Canada, namely 779.31 μg/g. For the cultivars ”Nimfa”, ”Amphora”, ”Leningradskij Velikan”, and ”Indigo Gem”, the highest content of phenolic acids, especially hydroxycinnamic acids, including chlorogenic acids, was recorded [37]. In the same vein, Orsavová et al. showed that the phenolic composition, as well as vitamin content of LCF, varies depending on the origin, cultivars, and meteorological conditions. Eight cultivars of *Lonicera caerulea var. kamtschatica* Pojark, with different origins and ripening periods, were analyzed (70% ethanolic extracts). The conclusions were, as expected, that the chemical composition of LCF depends on the several abovementioned factors. LCF grown in the area with more sunshine (Lednice) presented higher levels of monomeric anthocyanins and vitamin C, compared to berries cultivated in the rainy area (Žabčice), which showed higher levels of flavonoids and total phenolics. Concerning the ripening time, early ripening ”Amphora” presented the highest anthocyanin and vitamin C levels in both cultivation areas, and the medium–early-ripening ”Fialka” cultivar had an impressive phenolic content in both cultivation areas. In contrast, the ”Amphora” cultivar showed increased phenolic levels only in the Lednice area. Chlorogenic acid was present in good quantities in all cultivars, regardless of the locality of cultivation (2123.1 mg/kg–4770.8 mg/kg in Lednice cultivars; 2566.0 mg/kg–4654.9 mg/kg in Žabčice cultivars) [38].

The chemical composition of LC varieties can also fluctuate based on the altitude. Boyarskih et al. studied the leaves of the species *Lonicera caerulea var. altaica* from the Altai Mountains, Asia. The leaf extract was obtained using 70% ethanol in a water bath and HPLC-MS analysis was used to identify the individual components of the analyzed extracts. The main compounds in the leaf extracts of *Lonicera caerulea* var. *altaica* varied due to the difference in altitude. Hydroxycinnamic acid derivatives (chlorogenic and dicaffeoylquinic acids) ranged from 1176 to 3216 mg/100 g, flavonols (quercetin glycosides) from 342 to 1442 mg/100 g, and flavones (luteolin and apigenin glycosides) from 757 to 1988 mg/100 g. It was observed that flavone levels had positive correlations with the increase in altitude, while the content of flavonols decreased with increasing altitude [39].

Moreover, it was shown that the solar radiations, the temperature, and the precipitations have a major impact on the vitamin C content [38], with the remark that low temperatures and high humidity increase the level of ascorbic acid [40]. A study conducted by Jurikova et al. showed that the ascorbic acid level from methanolic LCF extracts, with berries collected from Slovakia (Central Europe) and Pavlovsk (Western Russia), can exceed concentrations ranging from 40.46 mg/100 g to 187 mg/100 g compared to other fruits with a high vitamin C content [41], such as oranges (31 mg/100 g), red currants (35 mg/100 g to 90 mg/100 g), elderberries (approximately 30 mg/100 g), and raspberries (16 mg/100 g to 32 mg/100 g) [42]. Viskelis et al. demonstrated that the vitamin C content is higher in northern countries than in southern ones [43]. Furthermore, Rupasinghe et al. emphasized in their 2018 review the superior abundance of vitamin C in haskap cultivars (ranging from 29 to 187 mg/100 g), compared to other vitamin C-rich sources such as strawberries (58.8 mg/100 g FW), blackberries (21 mg/100 g FW), raspberries (26.2 mg/100 g FW), and oranges (53.2 mg/100 g FW) [30]. Vitamin C is vital for health because it has antioxidant action and has been proven to have anti-inflammatory and anti-atherosclerotic effects [44].

The abovementioned information showed that the chemical composition of the species of LC depend on the type and duration of the extraction, the climatic conditions, the altitude, and its varieties harvested from different regions [26,28,39]. On the strength of the obtained results, C3G, rutin, and chlorogenic acid are the key phytochemical markers of LC extracts. This aspect is of particular importance for a possible standardization of LCF phytopreparations. At the same time, the presented aspects regarding the variable chemical composition of LCF bring to the fore aspects that concern the selection of the most valuable cultivars and the best pedo-climatic cultivation conditions. Due to its abundance of polyphenolic compounds and vitamin C, blue honeysuckle may possess greater health benefits than other edible berries. This aspect has sparked a new wave of research interest in studying the potential of *Lonicera caerulea* L. as a new functional food [11,45].

### 1.5. Pharmacological Activities, Cardiovascular Diseases and Metabolic Syndrome

Nowadays, the utilization of LCFs is increasingly gaining popularity in Europe, particularly in Poland, Slovenia, the Czech Republic, and Slovakia, owing to their significant medicinal attributes. Honeysuckle fruits are often utilized as a component of nutritional supplements and pharmaceutical formulations [11].

The Ainu aboriginal people of Japan have long recognized the curative properties of haskap fruits [46]. According to popular belief, it has been historically employed in traditional medicine as a means to potentially mitigate the likelihood of developing hypertension, glaucoma, heart failure, anemia, malaria, osteoporosis, and digestive disorders [10,30,47]. A phytochemical composition rich in polyphenols has numerous benefits for the human body, including anti-inflammatory and antioxidant activity, with major conveniences in chronic diseases [48,49]. The health-enhancing characteristics of haskap berries have been examined through in vitro and in vivo studies. These properties encompass safeguarding effects against cardiovascular, metabolic, and neurodegenerative ailments, osteoporosis, type 2 diabetes (T2DM), and anemia, in addition to antimicrobial, anticarcinogenic, and anti-inflammatory activity [10,11,50,51,52].

Cardiovascular diseases (CVDs) include a wide range of illnesses that impact the coronary blood arteries and the heart [53], being the leading cause of premature death and disability and thus, a public health concern among non-communicable diseases with huge socio-economic consequences [54]. They account for 37% of the deaths globally (around 20.5 million deaths) [55], a number that is projected to increase up to 32.3 million in 2050 [56]. CVDs encompass a wide range of conditions including arterial hypertension, lipid imbalances, cardiomyopathy, heart failure, coronary artery disease, and peripheral vascular disorders [57]. Arterial hypertension is the predominant cause of CVDs [58], affecting 33% of adults (age: 30–79) [59], worldwide, with women being the most affected [60]. Moreover, women exhibit greater death rates along with worse prognoses after acute cardiac incidents, such as a heart attack, coronary artery disease, cardiac failure, and aortic illnesses, as compared to males [61]. Most often, CVDs are associated with metabolic syndrome (a syndrome caused by modern life habits, consisting of a chronic-low grade inflammatory state, oxidative stress, hemodynamic dysfunction, and ischemia) which includes dysglycemia, high blood pressure, abdominal obesity, low high-density lipoprotein levels, and high triglycerides levels [62]. Each of these conditions, solely, is a risk factor for CVDs, but when combined (≥3 of them) for the same patient, it enhances the chances of developing cardiovascular and cerebrovascular complications, such as myocardial infarction and stroke (the first and third leading cause of death, globally) [63].

In the last decade, although the pharmaceutical industry has developed several efficient multi-target classes of medicines for cardio-metabolic diseases (i.e., SGLT-2 inhibitors and incretin mimetics), they are responsible for severe side effects and risk of interactions with other drugs and require high costs of treatment, aspects that limit their utilization. Therefore, new effective, accessible and less toxic pharmacological solutions are required for the so called “diseases of modern life” [64,65,66,67].

Thus, the purpose of the present systematic review (the research question) was to present the latest advances in the field regarding LCF’s ability in reversing cardio-metabolic imbalances.

## 2. Results and Discussion

### 2.1. Antioxidant Activity

It is well known that both pathologies, CVDs and metabolic syndrome, induce a multi-organ dysfunction, known as cardio-metabolic syndrome, having a prevalence of epidemic proportions [62]. Predisposing variables include detrimental behaviors such as smoking, excessive consumption of saturated fat and cholesterol, diabetes, and a lack of physical exercise [68]. These risk factors lead to changes in the integrity of blood vessels, decreased integrity of cell membranes, increased production of reactive oxygen species (ROS), and thus, a decrease in the body’s internal antioxidant system, culminating in oxidative stress [69]. Substances that possess the capacity to preserve the structural integrity of blood vessels and inhibit or minimize the generation of free radicals have the potential to be effective in the treatment of cardio-metabolic diseases, progressively being embraced by both the general population and healthcare practitioners [70,71]. Studies have shown that some compounds found in plants have properties that may protect the multi-organ dysfunction caused by CVDs and metabolic syndrome. These phytochemicals have antioxidant and anti-inflammatory effects, as well as the potential to shield the inner lining of blood vessels, prevent the oxidative breakdown of lipids, and enhance the body’s natural antioxidant defenses. Therefore, the significant functions of phenolics and flavonoids in therapeutic plants have been well-documented [70,72,73].

In recent years, extensive research has been conducted on the antioxidant capacity of extracts derived from LCF. In the short communication article published in 2012, Rupasinghe et al. demonstrated that honeysuckle berries exhibit greater activity compared to strawberries or blackberries, which are commonly consumed. Based on the findings of the ferric-reducing ability of plasma (FRAP) analysis, it was determined that the antioxidant capacity of cultivar ”Borealis” was 46.38 μmol TE/g fresh weight (FW). In comparison, strawberries exhibited an antioxidant capacity of 8.00 μmol TE/g FW, while blackberries showed a value of 15.03 μmol TE/g FW [74]. According to their bioactive component content and extraction technique, the antioxidants present in LCF participate in pathways that modulate oxidative stress [10,37]. Lee et al. demonstrated that LCF extract has a propensity to elevate micro-ribonucleic acid (mRNA) levels of genes involved in antioxidant activity, including heme oxygenase-1 (HO-1), NAD(P)H dehydrogenase [quinone] 1 (Nqo1), and glutamate-cysteine ligase catalytic subunit (Gclc), in HepG2 cells [75] (Table 1). It is known that nuclear factor E2-related factor 2 (Nrf2) activation may stimulate the expression of the aforementioned anti-oxidant genes [76]. In addition, LCF extract has shown an enhancement in the activity of antioxidant enzymes, including superoxide dismutase (SOD) and catalase (CAT), when tested on the HepG2 hepatocyte cell line [75].

Molina et al. investigated the nutritional value, as well as the chemical composition, of an extract obtained from haskap berries (*Lonicera caerulea* L. var. *kamtschatica*). The haskap berries were rich in water, organic acids (i.e., citric, malic, and quinic), free sugars (i.e., fructose and glucose), tocopherols (i.e., α- and γ-tocopherol) and fatty acids (in particular linoleic acid). The antioxidant capacity of the extract was assessed through thiobarbituric acid reactive substances (TBARSs) and oxidative hemolysis inhibition (oxHLIA) assays, highlighting a good antioxidant activity and no cytotoxicity [77] (Table 1).

Gawroński et al. assessed the phytochemical characteristics of 30 blue honeysuckle genotypes, including their antioxidant activity (through 2,2-diphenyl-1-picrylhydrazyl (DPPH) and 2,2′-azino-bis(3-ethylbenzothiazoline-6-sulfonic acid) (ABTS) techniques), in order to choose the proper ones with high nutraceutical value, highlighting the importance of breeding studies for the content boosting of biologically active substances (with health-promoting properties) [78] (Table 1).

The group of Česonienė et al. evaluated the bioactive substances, DPPH antioxidant activity and antibacterial potency of 11 cultivars of blue honeysuckle (ethanolic and aqueous extracts). The total phenolic content of ethanolic extracts fluctuated from 364.02 ± 0.41 mg/100 g in the ”Vostorg” cultivar to 784.5 ± 0.3 mg/100 g in the ”Obilnaja” cultivar. Moreover, the anthocyanin content (representing 53.8% of the total phenolic content) of the same ethanolic extracts varied from 277.8 ± 1.1 mg/100 g in the ”Čelnočnaja” cultivar to 394.1 ± 8.4 mg/100 g in the ”Nimfa” cultivar. The best antioxidant activity was exhibited by the ”Vostorg” and ”Eisbar” cultivars (respectively, 377.3 ± 8.5 mg TE/100 g FW and 371.8 ± 5.4 mg TE/100 g FW). The malic acid from the aqueous extracts exhibited good antibacterial activity on *Staphylococcus aureus*, *Escherichia coli*, and *Pseudomonas aeruginosa*, with no antibacterial activity observed for ethanolic extracts, thereby suggesting the health-promoting properties of blue honeysuckle cultivars [79] (Table 1). The authors concluded that the variations in the bioactive content between the cultivars has a direct impact on the intensity of the biological actions, highlighting the importance of properly choose the most valuable genotypes (highly rich in bioactive compounds) [79]. No toxicity assessment was performed.

In the article published in 2023, Fan et al. characterized the anthocyanins from 61 different genotypes of blue honeysuckle from northeast China, as well as their ROS scavenging activity, concluding that cyanidin-3,5-diglucoside has the highest ROS scavenging activity [80] (Table 1). This study highlighted as well the importance of genotype selection in the production of functional foods.

### 2.2. Hypolipidemic Activity

Hyperlipidemia is a very significant contributory factor for cardiovascular conditions as well as for metabolic imbalances [81]. Hyperlipidemia is associated with several disorders that pose significant risks to human health, including arteriosclerosis, coronary artery disease, heart attack, stroke, nonalcoholic steatohepatitis (NASH), microvascular diseases, etc. [82]. Numerous studies have demonstrated the efficacy of LCF in the treatment of metabolic imbalances, including obesity and diabetes [83,84,85,86].

Sirtuins, a group of enzymes that change proteins and rely on nicotinamide adenine dinucleotide (NAD+), have a significant impact on important biological processes such as glucolipid metabolism, oxidative stress, repair of deoxyribonucleic acid (DNA), and an inflammatory reaction. They have an impact on cardiovascular conditions such as atherosclerosis, myocardial infarction, hypertension, and heart failure. Research indicates that metabolic and bioenergetic reprogramming plays a crucial role in regulating inflammation, especially macrophages, serving as critical cells and producers of inflammatory cytokines [87]. Liu et al. demonstrated that the ethanolic extract of LCF, containing C3G, (+)-catechin, and chlorogenic acid, at a concentration of 80 μg/mL, activated sirtuin 1 in Raw264.7 macrophage foam cells. In addition, the extract induced a decrease in microRNA 33 (miR-33) and sterol regulatory element-binding protein 2 (SREBP2) expression, increased adenosine triphosphate-binding cassette transporter 1 (ABCA1) expression, decreased the macrophage cholesterol content, and suppressed the proliferation of Raw264.7 macrophage foam cells [88] (Table 1). The authors concluded that the study provides new insights for a novel hypolipidemic mechanism of LCF, involving the regulation of several substances with unelucidated mechanisms of action [88].

Probiotic fermentation is a very successful method for producing functional foods with hypolipidemic properties. Recent research studies have shown that fermenting medicinal herbs could strengthen their hypolipidemic efficacy. As a result, Luo et al. were able to demonstrate that the combined fermentation of *Lactobacillus casei* and *Bifidobacterium bifidum* is likely to have the most favorable impact on enhancing the hypolipidemic properties of LC berry juice. After undergoing fermentation with the ideal proportion of lactic acid bacteria, the rate of inhibition of pancreatic lipase was 63.55%, and the binding capacity of bile acid salt was 2.55 mg/mL. Upon subjecting HepG2 cells to oleic acid treatment, the low-density lipoprotein–cholesterol (LDL-C) levels exhibited a reduction of 41.43%, while the high-density lipoprotein–cholesterol (HDL-C) levels saw a rise of 61.36% (*p* < 0.05). These changes were seen when the fermentation broth concentration reached 10%. The content of total cholesterol (TC) and triglycerides (TG) in HepG2 cells fell progressively as the fermentation broth concentration increased, exhibiting a dose-dependent relationship [89].

In line with a recent investigation, the inhibitory effect of LC polyphenols on the translocation of lipopolysaccharides (LPS) is achieved through the modulation of both the microbiota of the intestine and the epithelial barrier of the intestine [90] (Table 1). Thus, Li et al. suggested that LCF could repair intestinal structure and function [90]. In a study conducted by Kim et al. it was discovered that extracts obtained from LCF demonstrated anti-obesity properties in mice that were fed with a high-fat diet (HFD). This effect was observed to be mediated through the activation of adenosine monophosphate-activated protein kinase (AMPK) [91] (Table 1). Research has demonstrated that polyphenols possess the ability to inhibit the absorption of lipids and mitigate weight gain [92,93]. In the most recent research conducted by Wang et al., it was found that the extract obtained from LCF, containing anthocyanins, exhibited a notable reduction in serum TG, TC, and LDL-C levels in HFD Sprague Dawley rats. Additionally, the extract was observed to increase the levels of fecal sterols in these rats. The administration of LCF demonstrated the ability to mitigate the impairment of the epithelial barrier in the small intestine. This effect was achieved by reducing the oxidative stress induced by the Nrf2-antioxidant response element (ARE) pathway and regulating the expression levels of various pro-inflammatory factors, including tumor necrosis factor-α (TNF-α), interleukin-6 (IL-6), cyclooxygenase-2 (COX-2), nuclear factor kappa-B p65 (NF-κB p65), and inducible nitric oxide synthase (iNOS) in the small intestine of subjects [84] (Table 1). Thus, the article highlights again the LCF’s involvement in maintaining small intestinal integrity, as well as a healthy intestinal microbiota. Moreover, LCF seems to be involved in blocking the intestinal absorbtion of fats [84]. Dayar et al. examined the potential benefits of LCF extract, as a potential source of polyphenols with therapeutic properties for cardiometabolic disorders. The researchers conducted their investigation using Zucker rats, an established animal model for obesity. The findings of the study indicate that LCF have the ability to lower levels of LDL and TC. This effect was accompanied by an upsurge in NOS activity and SOD expression, as well as a decrease in nicotinamide adenine dinucleotide phosphate (NADPH) oxidase and NF-κB. Consequently, the authors hypothesized that the enhanced lipid profile resulting from LCF consumption is due to the phytochemicals’ antioxidant effect [94] (Table 1) and that LCF supplementation could be beneficial in targeting cardiometabolic disturbances.

### 2.3. Hypoglycemic Activity

Diabetes mellitus (DM) is a group of metabolic illnesses characterized by consistently high levels of blood sugar [95]. It is linked with vascular disease, which leads to significant morbidity and mortality. Approximately 50% of individuals with DM have cardiovascular problems, resulting in death [96,97]. The metabolic imbalance results in significant damage to cellular and organ structures, particularly impacting the blood vessels and nerve fibers [96]. In recent decades, there has been significant interest in the potential benefits of anthocyanins for both the prevention and treatment of cardiometabolic diseases [98]. The available epidemiological evidence suggests that the inclusion of foods rich in anthocyanins in one’s diet may potentially reduce the likelihood of developing T2DM, hypertension, and CVDs [99,100,101]. The scientific literature recognizes that LCF extract has anti-diabetic properties by inhibiting the enzyme activities of *α*-amylase, *α*-glucosidase, and dipeptidyl peptidase-4, the intensity of their pharmacological actions depending on the cultivar type and harvesting date. Additionally, it may also prevent the production of late glycosylation end products [9,102,103]. Moreover, another study has shown that the antidiabetic effects of LC berries vary depending on the form in which they are utilized, such as powdered fruit, pomace, juice, and sugar-free juice products (SFJP). The predominant amount of flavan-3-ols, including around 48% of all quantifiable phenolics, was found in whole fruits and juice powders. This was accompanied by anthocyanins at 24%, phenolic acids at 15%, flavonols at 8%, and procyanidins at 5%. Concerning the phytoconstituents that were identified in the pomace and SFJP were ranked in the following order: anthocyanins > flavan-3-ols (monomers and dimers) > procyanidins > phenolic acids > flavonols. The *α*-glucosidase inhibiting activity of the haskap berry products was evaluated, and it was found that the SFJP (IC_50_ [half maximal inhibitory concentration] = 0.29 mg/mL), pomace powders (IC_50_ = 5.35 mg/mL), and whole fruit powders (IC_50_ = 5.57 mg/mL) exhibited the most potent inhibitory activities. The presence of anthocyanins and procyanidins in haskap berry powders showed a significant correlation with the capacity to inhibit *α*-amylase. On the other hand, phenolic acids had a more pronounced impact on the capacity to suppress *α*-glucosidase. The research group also showed that the composition of amino acids affects anti-diabetic efficacy. A significant inverse relationship was observed regarding the glycine ratio and the capacity of haskap berry to inhibit *α*-glucosidase activity. While glycine did not constitute the most prevalent amino acid, this finding implies that the fruit matrix influences the selectivity of an amino acid’s action on the enzymes under investigation [104] (Table 1).

Substantial data indicate a strong correlation between the development of T2DM and the composition of the gut microbiota. Cao et al. aimed to assess the effect of an ethanolic extract derived from LC berries, which were inoculated with *Lactobacillus rhamnosus*, on the inhibitory activity of α-amylase and *α*-glucosidase. The fermented sample exhibited 82% inhibition of *α*-amylase at a concentration of 4 mg/mL, while the unfermented sample showed 71% inhibition. In a comparable fashion, the *α*-glucosidase inhibitory rate showed a similar trend. The fermented sample exhibited a higher inhibitory rate (94%) compared to the unfermented group (90.2%) at the same dose. Furthermore, LCFs successfully restored the dysbiosis of the intestinal microbiota induced by T2DM, as shown by an augmentation in the prevalence of microorganisms such as *Lactobacillus*, *Blautia*, and *Bacteroides* [105], thus suggesting again the interplay between gut microbiota and LCF (and its increased medicinal potential) which needs to be confirmed by in vivo studies.

Yang et al. performed a comprehensive review and meta-analysis of randomized controlled trials investigating the impact of anthocyanins on lipid profiles and glycemic management in populations with and without cardiometabolic disorders. The results of this study indicate that anthocyanins have a significant impact on various markers of glucose and lipid metabolism. Specifically, fasting glucose levels were found to be significantly reduced (standardized mean difference [SMD]: −0.31; 95% confidence interval [CI]: −0.59, −0.04; I^2^ = 80.7%). Similarly, postprandial glucose levels at 2 h after a meal showed a significant decrease (SMD: −0.82; 95% CI: −1.49, −0.15; I^2^ = 77.7%). Moreover, glycated hemoglobin (HbA1c) levels, a long-term indicator of glucose control, were significantly reduced (SMD: −0.65; 95% CI: −1.00, −0.29; I^2^ = 72.7%). In addition, anthocyanins were found to have a significant impact on TC levels (SMD: −0.33; 95% CI: −0.62, −0.03; I^2^ = 86.9%) and LDL levels (SMD: −0.35; 95% CI: −0.66, −0.05; I^2^ = 85.2%) [106].

In a study conducted by Sharma et al., mice fed with a HFD exhibited elevated levels of insulin, blood glucose, HbA1c, blood urea nitrogen, and creatinine. Furthermore, the rats exhibited a higher quantity of degenerative lesions and pancreatic islet cells compared to the control group, which affects the release of insulin and glucagon. When rodents were fed with honeysuckle berry extracts, the aforementioned complications diminished significantly. In contrast to mice fed with a HFD, mice fed with 400 mg/kg extracts experienced a decreased risk of T2DM and other positive effects such as the amelioration of complications associated with diabetic nephropathy. The study also evaluated the dose-dependent activity of honeysuckle berries, indicating their potential as a diabetes treatment food [51]. Chun et al. conducted a study to examine the impact of consuming LCF extract on obese mice with mild diabetes who were fed with a HFD. The findings of the study demonstrated a notable reduction in body weight and a decrease in adipose tissue in the abdominal wall and periovarian region, providing evidence for the anti-obesity properties of LCF [107] (Table 1). Additional research has corroborated the presence of certain components within LCF extracts that possess anti-diabetic properties, resembling the effects of insulin [108]. These components have been observed to impede the breakdown of starch in the gastrointestinal tract. Furthermore, it was observed that they exerted a direct stimulatory effect on the production of insulin [108].

According to a recent study, it was found that LCF extracts exhibit anti-sarcopenic obesity properties in mice that were fed with a HFD. It is worth noting that sarcopenic obesity translates to a decreased mass and muscle strength in skeletal muscles, leading to excessive body fat, which will later induce insulin resistance (by interfering with mitochondria’s oxidative capacity). Therefore, sarcopenic obesity was associated with cardio-metabolic morbidity and mortality. The anti-sarcopenic obesity properties of LCF were observed through a reduction in body weight, adipocyte size, and abdominal and subcutaneous fat mass and through an increase in muscle mass (by regulating SIRT1 and PGC1α genes) [85] (Table 1).

The study performed by the group of Lee showed that C3G increased the secretion of insulin, therefore leading to a decrease in the production of glucose, in an INS-1 cell line (a model of pancreatic β cell), through an increase in insulin receptor phosphorylation and a decrease in insulin receptor substrate (IRS-1) and phosphoinositide 3-kinase (PI3K) protein expression [109].

### 2.4. Hepatoprotective Activity

Modern life habits, such as a sedentary lifestyle, increased stress, and high consumption of processed foods, are the potential aetiologic factors of the cardio-metabolic imbalances and multi-organ dysfunctions seen in today’s population [62]. The high caloric intake and the physical inactivity induce a state of insulin resistance and an increased body mass index (including a high waist circumference), with deleterious repercussions over the entire body, due to the state of low-grade inflammation and neurohormonal activation installed over time. Therefore, the cardio-metabolic syndrome is not a disease per se, but an ”umbrella” of risk factors (including genetic factors and aging), most of them being modifiable, with pathophysiological mechanisms that are far from being elucidated [65,110].

The lipid accumulation takes place also in the hepatocytes, where triglycerides are stored, triggering, at first, simple steatosis. In time, the high intake of lipids and carbohydrates will induce a state of lipotoxicity and glucotoxicity, with dramatic impact on the liver, more specifically inflammation and NASH, which can lead (if not managed) to hepatic fibrosis, in a subset of patients [111]. Therefore, substances that can mitigate the deleterious effects of fat infiltration in the hepatocytes, inducing a hepatoprotective effect (through the modulation of gut microbiota, attenuation of inflammation and of mitochondrial defects, and decreasing endoplasmatic reticulum stress or overall oxidative stress), can be of great value for the long-term management of syndrome X (known as metabolic syndrome) [112].

The group of Wu S. et al. published three papers (studies on animal models) regarding the protective effects of LC polyphenols obtained from berries on non-alcoholic fatty liver disease (NAFLD) [113,114,115]. Both NAFL and NASH are types of NAFLD. In the first study of a NASH-induced mice model (mice fed with a HFD containing *Lonicera* polyphenols 0.5–1% or not), the polyphenols managed to improve the histopathological features of NASH, and ameliorate inflammation and lipid peroxidation through the upregulation of Nrf2 and manganese-dependent SOD (MnSOD) and downregulation of forkhead box protein O1 (FoXO1) and HO-1 [115]. In the second published study, the experimental NAFLD induced in a mouse model was ameliorated through a reduction in obesity and hepatic fat deposition by the administered blue honeysuckle extract. The extract managed to improve insulin sensitivity and decreased oxidative stress through the upregulation of the Nrf2-mediated pathway, an important defense mechanism against oxidative stress and inflammation [114] (Table 1). The previous studies were continued with the evaluation of polyphenols’ actions on gut microbiota, in the same experimental mice model of fatty liver disease, as gut endotoxins are assumed to be the underlying cause of NAFLD inflammation. The 45 days supplementation with blue honeysuckle extract (0.5–1%) led to a serum decrease in IL-2, IL-6, monocyte chemoattractant protein-1 (MCP-1), and TNF-α, and to a serum and liver level decrease in endotoxins in HFD-fed mice. Moreover, the fecal microbiota was improved (the *Firmicutes*/*Bacteroidetes* ratio was ameliorated), all these results improving the scientific understanding of NAFLD pathogenesis, as well as the *Lonicera* polyphenols’ positive effects on this pathology [113].

The study published in 2016 by Wang et al. highlighted the protective effects of LCF extract against liver damage induced by LPS in a BRL-3A cell line model, before and after in vitro digestion on hepatitis. In both situations, LCF extract decreased oxidative stress, sustained cellular structure, and its metabolism, modulated liver function, and reduced the synthesis of pro-inflammatory cytokines (i.e., IL-1β and IL-6) [116] (Table 1). The same group published next year another study in which they showed the reduction in liver toxicity in the same cell line, through LCF antioxidant, anti-inflammatory, and anti-apoptotic actions [117] (Table 1).

Park et al. investigated the biologically active properties of honeyberry extract addition on HepG2 cellular steatosis (induced by free fatty acids) in obese mice. The administered extract inhibited fatty acid synthesis and accumulation in HepG2 cells, as well as diminished lipid accumulation in the liver. It decreased triglyceride accumulation, thus highlighting its potential of reversing non-alcoholic fatty liver disease [118] (Table 1).

Also, Lee et al. examined the effect of blue honeysuckle water extract rich in C3G in inhibiting the adipocytes’ differentiation, cell line—3T3-L1. They showed that the blue honeysuckle extract managed to inhibit adipogenesis and to downregulate the expression of several transcription factors involved in the adipogenesis pathway, but not to affect lipolysis, remarking its potential as a natural anti-obesity candidate [119].

The anti-inflammatory effects of leaf and branch LC extracts were also evaluated in an LPS-stimulated RAW264.7 cell line by An Mi-Yun et al. They showed that both extracts induced an anti-inflammatory effect through the activation of activating transcription factor 3 ATF3 (having an important role in the innate immunity, its expression decreasing the production of several inflammatory mediators) and Nrf2/HO-1 (a redox-sensitive transcription factor which regulates the expression of antioxidant enzymes). The anti-inflammatory effects of leaf and branch blue honeysuckle extracts were stronger than those observed for the fruit extract [120].

The anti-obesity potential of LCF was also explained by the inhibition of lipid accumulation in adipocytes through lipogenesis suppression via AMPK activation. The primary polyphenols (flavonoids, mainly flavonols and anthocyanins) were incriminated for the dose-dependent effect. They repress lipogenesis by enhancing the phosphorylation of AMPK and by reducing the expression of lipogenic transcription factors. Moreover, they can increase the markers of beige adipose cells [121].

In 2023, the group of Shao published a study where two selenized polysaccharides obtained from a native polysaccharide of LCF fruit were synthesized, characterized, and biologically evaluated. The two selenized polysaccharides (PSLP-1 and PSLP-2) induced a higher antioxidant activity than the native one, but their bile acid-binding capacity and acetylcholinesterase inhibitory activity decreased, thus elucidating the advantages of selenylation and polysaccharides’ use as potential therapeutical solutions [122] (Table 1).

### 2.5. Vasoprotective Activity

As previously indicated, LCF (cultivars ”Wojtek”, ”Leningradskij Velikan, Wild”, ”Beilei”, ”Amphora”, ”Amur”, ”Jolanta”, ”Kuvshinovidnaya”) contain a significant amount of C3G [30,48,123]. Research undertaken in laboratory settings has shown that C3G and its metabolites enhance the activity of vascular endothelial NOS (eNOS), hence improving endothelial function [124,125] (Table 1). Furthermore, C3G has shown an increase in the activation of genes and proteins that protect against oxidative stress, which is believed to be facilitated by the production of Nrf2. This increase is connected with the maintenance of muscle function after intense resistance exercise [126]. Based on these hypotheses, Howatson et al. performed a research investigation employing a double-blind, placebo-controlled trial to evaluate the impact of freeze-dried powder from LCF on well-established and commonly used measures of endurance running performance. The study included 30 recreational runners. The findings indicate that reduced heart rate (HR) and oxygen consumption (VO_2_) were seen during low-intensity exercise (lactate threshold). Additionally, there was a significant benefit of around 2.2% in running efficiency time to tiredness with the acute ingestion of haskap during the VO_2peak_ test, lasting around 20 s. The minor impacts were reflected in an enhanced 5 km time trial performance, with an approximate improvement of 21 s (equivalent to a 0.25 km/h increase in average running speed). This modification has significance within the realm of human running capabilities [127]. A recent study recognized the favorable impacts of LCF as a nutritional agent that may be regarded as an alternative for alleviating exercise tiredness. Male BALB/c mice were administered an intragastric dose of LCF ethanolic extract at a concentration of 250 mg/kg body weight, one hour prior to being subjected to treadmill activity. Supplementing the diet with LCF notably increased the exhaustion time during treadmill activity by 20.4% at 25 °C and 27.4% at −5 °C. The administration of LCF resulted in enhanced energy storage, specifically in the liver glycogen and muscle glycogen. It also decreased the buildup of metabolic byproducts, reduced oxidative stress and inflammatory responses, prevented cell death in skeletal muscle cells, stimulated cell growth, mitigated muscle damage caused by prolonged exercise fatigue, reduced fatigue, and ultimately improved athletic performance. The primary processes implicated were the decrease in reactive oxygen species and the activation of the mitochondrial apoptotic pathway, which provided safeguarding to skeletal muscle [128] (Table 1).

The health benefits of LCF extract were documented in a human study conducted in the Czech Republic. The study involved 639 healthy individuals. These participants consumed a quantity of berries equal to 165 g per week. Results showed a notable increase in the levels of glutathione peroxidase (GPx) and CAT in both erythrocytes and plasma. Importantly, no negative effects were observed as a result of this berry consumption. A double-blind, crossover intervention study involving 20 healthy elderly participants revealed an important beneficial impact of honeysuckle berry extract on physiological and cognitive abilities in the near-postprandial period. The administration of a 400 mg dose was observed to cause a reduction in diastolic blood pressure and HR within a relatively short period of 1.5 h [129]. This observation was correlated to the previously investigated effects of anthocyanins on vasodilation and glucose regulation [10]. The aforementioned physiological alterations can be linked to enhanced cognitive function, which is achieved by an augmentation in cerebral blood flow and an elevation in glucose uptake by the brain. The administration of a high dose (400 mg) of LCF extract was found to lead to a substantial enhancement in episodic memory. Additionally, it has been proposed that doses lower than 100 mg are inadequate in terms of their impact on episodic memory. According to the outcomes of the study, honeysuckle berries have tremendous potential for preventing age-related memory loss and improving metabolic and vascular health [129].

Inflammation is an intricate physiological reaction of blood vessels to detrimental stimuli. The process of inflammation includes the activation of cells and the production of substances known as inflammatory mediators [71]. Inflammation activation has been identified as a significant mechanism in the development and advancement of CVD, such as progressive heart failure. Interleukins and cytokines have been shown to induce inflammation in the artery wall [130]. Additional variables that contribute to inflammation include leukocyte adhesion molecules and chemokines [131]. Risk factors, such as elevated cholesterol levels, may cause an inflammatory response in the small blood vessels [10]. This is evident via the activation of endothelial cells, recruitment and attachment of white blood cells, and activation and attachment of platelets [132,133]. This may be bypassed by ensuring an adequate supply of nitric oxide in the blood vessels. However, a decrease in the presence of nitric oxide leads to impaired blood vessel function and reduced anti-inflammatory characteristics of the endothelium [134]. The anti-inflammatory effect of Korean and Chinese LCF methanolic extract was reported by An et al. According to the research, a reduction in nitric oxide production was detected in RAW 264 macrophage cells subsequent to treatment with 100 or 300 µg/mL of Korean and Chinese LCF [120]. In 2015, Rupasinghe et al. proposed that the anti-inflammatory activity of LCF methanolic extracts from Canadian cultivars (”Berry Blue”, ”Borealis”, ”Tundra”, and ”Indigo Gem”) may be attributed to the inhibitory effects on cytokines TNF-α, IL-6, PGE2, and COX-2 in LPS-stimulated THP-1 derived human macrophages. The observed effect was similar to the efficacy of diclofenac, a non-specific COX inhibitor drug. Out of all the other types of crops, Borealis consistently showed the most inhibiting effects. Borealis cultivar, at a dosage of 100 μg/mL, had dose-dependent effects on inflammation by suppressing the production of cytokines TNF-α, IL-6, prostaglandin E2 (PGE2), and COX-2 by 55%, 50%, 52%, and 38% correspondingly [135]. Another in vitro study conducted by Sanjay et al., demonstrated that carbon quantum dots obtained from LCF may suppress the mRNA levels of proinflammatory cytokines (TNF-α, IL-1β, and IL-6) while enhancing the production of anti-inflammatory cytokines (IL-4, IL-10, and transforming growth factor β (TGFβ)) in human microglial cells (HMC3) induced with LPS [136] (Table 1). Mice that were fed a diet rich in fat and induced with NASH had a decline in the release of proinflammatory cytokines, instead of an upsurge in the synthesis of anti-inflammatory cytokines after oral treatment with either 0.5% or 1% of an ethanolic extract derived from LCF [113]. Wu et al. proposed a dual modulation of LCF polyphenols in LPS-induced inflammation. On the one hand, the extract reduced serum inflammatory markers, such as IL-4, IL-6, IL-10, IL-12 (p-70), macrophage inflammatory protein-1α (MIP-1α), MCP-1, and TNF-α in the RAW264.7 cell line, as well as in ICR mice serum, after paw edema induction. On the other hand, the extract rich in polyphenols sharpened the expression of Nrf2 and MnSOD, thus inhibiting the inflammatory process through an antioxidant response [137] (Table 1).

**Table 1:** Main characteristics of the included studies reporting LCF’s antioxidant effect.

Study Team & Year	Type of Extract	Type of Assessment (In Vitro/In Vivo)	Method(s)	Results	Reference
Lee et al., 2018	Korean and Chinese lyophilized LCF juice	HepG2 hepatocyte cell line	Radical scavenging (DPPH assay) SOD and CAT activity ARE luciferase activity Nrf2-dependent antioxidant gene expression by real-time PCR	The radical scavenging activity increased in a concentration-dependent manner for both tested juices. At the highest tested concentration (300 μg/mL), Korean LCF juice exhibited 86.1% activity, while Chinese LCF juice showed 92.96% activity. Regarding SOD and CAT evaluation, both enzyme activities were increased by treatment with 300 μg/mL of each juice ([SOD activity]: Korean LCF juice—25.7 U/mg protein; Chinese LCF juice—33.7 U/mg protein; [CAT activity]: Korean LCF juice—506.5 μM/mg protein; Chinese LCF juice—802.2 μM/mg protein). ARE-driven luciferase activities were increased concentration-dependent, Korean LCF juice exhibiting the most vigorous induction of enzyme activity (3.9 fold at 300 μg/mL). mRNA levels of Nqo-1, HO-1, and Gclc were increased after treatment with both tested juices at 300 μg/mL. No in vitro toxicity was observed.	[75]
Molina et al., 2019	*Lonicera caerulea var. kamtschatica* (Wojtek cultivar) lyophilized powder	-	TBARS assay OxHLIA assay	Haskap fruit extract (80% hydroethanolic extract) showed an important antioxidant activity (TBARS: IC_50_ = 29.9 ± 0.3 μg/mL; OxHLIA: IC_50_ = 145 ± 5 μg/mL after 60 min, respectively IC_50_ = 938 ± 49 μg/mL after 120 min). The antimicrobial capacity was also evaluated (on six bacteria and six fungi), as well as cytotoxicity assessment.	[77]
Gawroński et al., 2020	30 genotypes of LC (extracts–acidified (0.1% (*v*/*v*) formic acid) 80% (*v*/*v*) methanol)	-	DPPH assay ABTS assay	1-17-59 and Amphora genotypes showed the best antioxidant activities. 1-17-59 genotype: 2.2 ± 0.01 mmol TE/100 g FW (DPPH) and 5.5 ± 0.17 mmol TE/100 g FW (ABTS). Amphora genotype: 2.0 ± 0.03 mmol TE/100 g FW (DPPH) and 4.5 ± 0.28 mmol TE/100 g FW (ABTS).	[78]
Česonienė et al., 2021	11 cultivars of LC (extracts–ethanol 95% (*v*/*v*) acidified with 0.1 N HCl)	-	DPPH assay	The Vostorg cultivar delivered the best antioxidant activity (377.3 ± 8.5 mg TE/100 g FW), closely followed by the Eisbar cultivar (371.8 ± 5.4 mg TE/100 g FW).	[79]
Fan et al., 2023	61 genotypes of LC (anthocyanin extraction – acidified methanol (0.1% (*v*/*v*) H_3_PO_4_ in 100% (*v*/*v*) methanol))	-	Superoxide anion radical scavenging activity (∙O_2_^−^) Hydroxyl radical scavenging activity (∙OH^−^)	Nine anthocyanins presented marked ROS scavenging activity, with cyanidin-3,5-diglucoside holding the best effect (with a correlation coefficient of 0.9413 for ∙OH^−^ and 0.9521 for ∙O_2_^−^).	[80]
Liu et al., 2019	LCF (polyphenol extraction–acidified ethanol (0.1% HCl (*v*/*v*) in 80% (*v*/*v*) ethanol))	RAW 264.7 macrophage cell line	siRNA and cell transfection Real-time PCR	Three main polyphenols, namely C3G, (+)-catechin, and chlorogenic acid, were found to activate SIRT1 at doses of 80 μg/mL, which further provoked a decrease in miR-33 and SREBP2 expression. At the same time, ABCA1 expression was increased. SIRT1 plays a major role in lipid and glucose metabolism regulation, as well as in oxidative stress modulation.	[88]
Li et al., 2020	LCF polyphenols (50% hydroalcoholic extraction)	Male Sprague Dawley rats	Oxidative stress-related indicators measurement in a rat model of oxidative stress damage	Significantly decreased levels of iNOS, MDA, and ROS were determined by LCF polyphenols, while levels of antioxidants, such as SOD and GPx were higher. Therefore, intestinal oxidative stress damage and lived damage could be mitigated also by activation of the Nrf2/HO-1/NQO1 and MAPK pathways.	[90]
Kim et al., 2018	LCF powder	Imprinting control region (ICR) mice	Lipid peroxidation and antioxidant defense system measurement in a HFD-induced model	Decreases in the antioxidant defense system (CAT, SOD, GSH), as well as an increase in lipid peroxidation MDA marker, were observed after a HFD. However, significant changes were noticed following treatment with LCF in doses of 100 mg/kg, 200 mg/kg, and 400 mg/kg. Liver MDA levels decreased with −29.82%, −44.13%, and −58.57%, respectively, in the LCF-treated groups, compared with the increase of 466.75% registered in the HFD control group. Regarding the antioxidant defense system, dose-dependent increases in CAT, SOD, and GSH were noticed after treatment with LCF (100, 200, and 400 mg/kg) in HFD mice.	[91]
Wang et al., 2023	LCF (anthocyanin extraction–acidified methanol (0.1% HCl))	Male Sprague Dawley rats	Oxidative stress-related indicators measurement (ELISA) in a HFD rat model	Eight-week supplementation with 250 mg/kg LCF determined a lowering in ROS and MDA levels, and an increase in SOD and GPx levels, compared to the HFD rats control group. Moreover, increases in cytoplasmatic Nrf2, HO-2, and Nqo1 were registered after LCF treatment in HFD rats. These findings suggest that LCF may counteract oxidative stress by Nrf2-ARE pathway modulation.	[84]
Dayar et al., 2021	LC dry fruits	Male obese (fa-/fa-) Zucker rats	Measurements of SOD, NADPH oxidase (Western Blot analysis), and conjugated diene (UV-Vis spectroscopy) in an animal model of cardiometabolic diseases	Six-week treatment with LCF induced an increase in SOD expression. In the same time, decreases in NADPH oxidase level, as well as in conjugated diene (a marker of lipid peroxidation) concentration were observed. Thus, the researchers concluded that the antioxidant effect of LCF could be responsible for the improvement in the lipid profile and for the enhancement of vascular NOS activity.	[94]
Brzezowska et al., 2023	LCF powder extracts (acidified methanol (0.1% HCl (*v*/*v*) in 80% (*v*/*v*) methanol)	-	TEAC ABTS and FRAP assays	Potent antioxidant activities were registered for all the tested samples (powdered fruit, pomace, juice, and SFJP). The best results were obtained for SFJP (ABTS: 273.27 ± 13.08 mmol Trolox/100 g DW; FRAP: 269.10 ± 1.36 mmol Trolox/100 g DW).	[104]
Chun et al., 2018	LCF aqueous extract	Female SPF/VAF CrljOri:C D1[ICR] mice	Oxidative stress-related indicators measurement in an HFD mice model	Intensive lipid peroxidation (transposed as high MDA content) and marked decreases in SOD, CAT, and GSH levels, were observed in the HFD control group. MDA level was significantly normalized after treatment with LCF extract for all the tested concentrations (100, 200, and 400 mg/kg). The endogenous antioxidant defense system was also markedly normalized after the applied treatment.	[107]
Lee et al., 2021	Raw LCF mixed with deionized water (containing 1% citric acid) at a ratio of 1:10 (*w/v*)	Male C57BL/6 mice	Oxidative stress-related indicator measurement (RT-PCR) in an HFD mice model	As expected, antioxidant enzyme expression was decreased in HFD control group. Treatment with 100, 200, and 400 mg/kg LCF extract significantly improved the expression levels of SOD, CAT, and GPx enzymes.	[85]
Liu et al., 2018	75% ethanolic LCF extract	Male C57BL/6N mice	Oxidative stress-mediated lipid peroxidation	Hepatic levels of Nrf2, MnSOD, and HO-1 were decreased by 60% in HFD control group, while supplementation with 0.5% and 1% LCF extract restored the levels of all antioxidant proteins. Withal, the lipid peroxidation process (expressed as TBARS level) was significantly decreased following treatment with LCF extracts.	[114]
Wang et al., 2016	LCF acidized methanolic extract (0.1% HCl) ± in vitro gastrointestinal digestion (digestive juice)–digested extract (DE) and undigested extract (UE)	BRL-3A rat liver cells	Oxidative stress-related indicators measurement in BRL-3A cells under LPS activation	Both DE and UE significantly impeded ROS production in LPS-activated cells, with an increased effectiveness observed for the pre-treatment with undigested extract. Intracellular glutathione level was increased following pre-treatment with the extracts, the differences between the DE and UE being significant (4.2 nmol/mg protein for UE, respectively 3.6 nmol/mg protein for DE). Further, DE and UE suppressed TBARS formation, thus the lipid peroxidation, decreased remarkably 8-OHdG and protein hydroxyls levels (UE exhibited stronger outcomes compared to DE).	[116]
Wang et al., 2017	LCF acidized methanolic extract (0.1% HCl) ± in vitro gastrointestinal digestion (digestive juice)–digested extract (DE) and undigested extract (UE)	BRL-3A rat liver cells	SOD, CAT, and total antioxidant capacity (T-AOC) measurements in LPS-activated BRL-3A cells	CAT, SOD, and T-AOC values were significantly increased after treatment with UE and DE. Thus, both extracts exerted an antioxidant action in LPS-induced oxidative stress. However, UE produced superior effects compared to DE, which may be due to a superior cyanidin-3-glucoside content.	[117]
Park et al., 2019	25% LCF ethanolic extract	ICR male mice	Oxidative stress-related indicators measurement in an HFD mice model	MDA levels were increased by 89% in the HFD group compared to the control group. In the same vein, SOD, GPx, and CAT levels were decreased in the HFD group. Six-week treatment with 0.5% LCF extract (LH) and 1% LCF extract (MH) determined an improvement in the oxidant-antioxidant balance. MDA levels were significantly reduced (HFD: 66.5 ± 11.4 nmol/g; LH: 51.1 ± 4.7 nmol/g; MH: 38.4 ± 4.4 nmol/g), while antioxidant enzyme values were markedly increased ([SOD] –HFD: 41.3 ± 2.2 μg/mL; LH: 54.8 ± 11.5 μg/mL; MH: 55.5 ± 4.1 μg/mL; [GPx] –HFD: 1.0 ± 0.2 μg/mL; LH = MH: 1.3 ± 0.2 μg/mL; [CAT] – HFD: 21.1 ± 1.0 ng/mL; LH: 25.3 ± 5.7 ng/mL; MH: 26.2 ± 2.7 ng/mL).	[118]
Shao et al., 2023	2 selenized polysaccharides (PSLP-1 and PSLP-2) extracted by enzymatic method from LCF (80% ethanolic extract)	-	ABTS assay BHT anti-lipid peroxidation assay AChE assay	PSLPs exerted increased antioxidant activity, in a dose-dependent manner (at 4.0 mg/mL the maximum scavenging potential was 28.85 ± 0.68% for PSLP-1, 39.37 ± 0.84% for PSLP-2, respectively 99.79 ± 0.20% for vitamin C used as positive control), compared with PLP—the native polysaccharide, suggesting the selenylation’s potential in improving the radical scavenging activity. Unfortunately, their acid-binding abilities and inhibitory activity on AChE decreased.	[122]
Kucharska et al., 2017	27 cultivars and 3 genotypes (80% methanolic LCF extracts)	-	FRAP assay DPPH assay Trolox assay TPTZ assay	The antioxidant activity varies largely between the cultivars, based on both iridoids and polyphenols’ amount. Strong correlation was found between anthocyanins content and antioxidant activity.	[123]
Liu et al., 2022	70% ethanolic LCF extract	Male BALB/c mice	Oxidative stress-related indicators measurement (DHE staining Fluorescence microscopy)	Exercise fatigue (in a treadmill endurance) at a low temperature (−5 °C) increased ROS production in skeletal muscle, while LCF dietary supplementation decreased it, as well as oxidative damage, through increasing NO production (downregulation of iNOS and upregulation of NQO1 and HO-1). LCF downregulated PKCα-Nox2/Nox4 pathway and upregulated AMPK-PGC1α-NRF1-TFAM axis in skeletal muskel, thus relieving exercise fatigue (most probably through cyanidin-3-glucoside, catechin, and chlorogenic acid’s synergic action).	[128]
Sanjay et al., 2023	Carbon quantum dots (CQDs) obtained by hydrothermal synthesis from fresh LCF	HMC3 human microglial cell line	DPPH assay ABTS assay Oxidative stress-related indicators measurement	CQD IC_50_ was 0.25 mg/mL, while at 1 mg/mL, the DPPH scavenging activity (91.2%) was proportional with ascorbic acid’s standard. For ABTS, CQD IC_50_ was 0.625 mg/mL, with a scavenging ability of 93.8%. Moreover, CQD decreased ROS level induced by LPS treatment in HMC3 cell line by increasing the expression of SOD, CAT, HO-1, HO-2, GPx, and Nrf2, probably by interfering with Nrf2/HO-1 signaling pathway.	[136]
Wu et al., 2017	75% ethanolic LCF extract	RAW264.7 mouse macrophage-like cell line	Oxidative stress-related indicators measurement	LCF enhanced the expression of Nrf2 and MnSOD in earlier response, without affecting the levels of HSP70 and iNOS. The fact that the induction time of the two enhanced markers began earlier (from 6 h) than that from LPS (12 h) suggests that LCF might activate the antioxidant response earlier, in order to counteract the oxidative stress produced by LPS.	[137]

**Figure 1:**
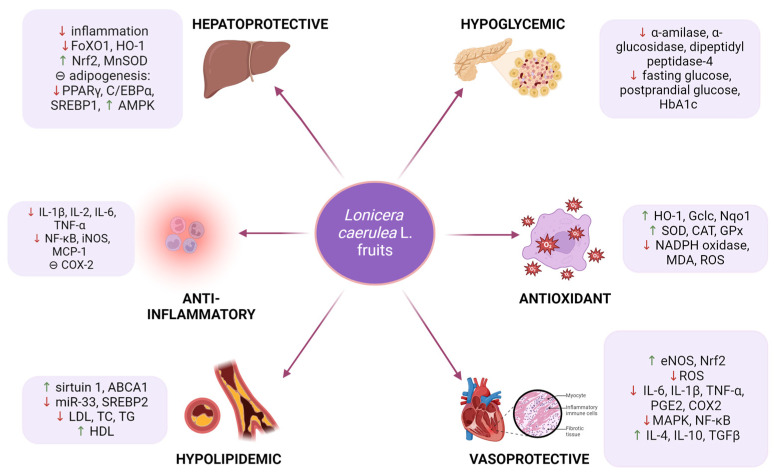
The main mechanisms of action described in the present review for the cardio-metabolic properties of *Lonicera caerulea* L. (↑—increase; ↓—decrease).

A summary of the main LCF’s mechanisms of action described in the present review are highlighted in Figure 1.

## 3. Materials and Methods

**Figure 2:**
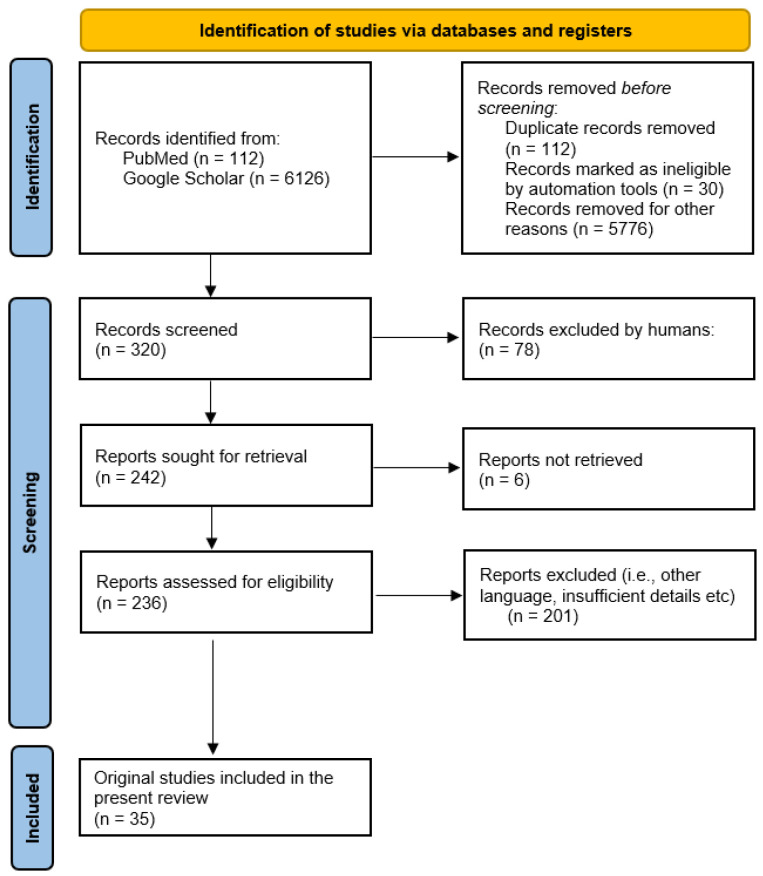
PRISMA flow chart of the present systematic review [139].

In order to assess the state of the art regarding the cardio-metabolic properties of *Lonicera caerulea* L., we aimed to conduct a systematic review of these pharmacological activities (PROSPERO Registration Number: CRD42024540002). Therefore, two researchers performed independent searches on PubMed (electronic database) and then Google Scholar (web search engine) from December 2023 to January 2024. The keyword combination consisted of *Lonicera caerulea* L. AND the searched pharmacological action (i.e., antioxidant, hypolipidemic/hypolipemiant, hypoglycemic/hypoglycemia, anti-diabetic, anti-obesity, hepatoprotective, or vasoprotective). Inclusion criteria: reviews and original articles (in vivo/in vitro studies) published from 1 January 2016 until 1 December 2023, assessing the mentioned pharmacological actions, either of the plant extract or isolated compounds of haskap, written in English. Exclusion criteria: articles that did not respect the inclusion criteria, as well as other publications that appeared to be methodologically flawed and provided insufficient details or confusing outcomes, book chapters, short communications, and letters (Figure 2). Another researcher removed the duplicates using an Excel sheet, based on the authors’ name, title, and publication year. Data extraction was performed by the previous two researchers who performed the initial research. Studies lacking a control substance/a comparator/a control group were removed to minimize the risk of bias. Eventual disagreements/misunderstandings were discussed with the entire team of the present article, in a face-to-face meeting, in order to reach a solution or a final conclusion regarding the inclusion/exclusion of an article in the present review (Figure 2). The protocol of the present systematic review was listed in PROSPERO Register (Registration Number: CRD42024540020).

Figure 1 was developed based on the aforementioned information in the present article using BioRender.com (accessed on 18 April 2024) [138].

This review includes articles that have not been discussed in the previously published reviews, namely the research of Golba et al. (2020) and Negreanu-Pirjol et al. (2023) [10,45].

## 4. Conclusions

The purpose of this comprehensive review was to give an in-depth up to date review of the most important and recent findings regarding the effects of *Lonicera caerulea* L.’s fruits on reversing cardio-metabolic disturbances. Although the review presents the most important pharmacological actions independently (by category), it is worth noting that each pharmacological action must be seen as a piece in the puzzle and that each pharmacological action is acting in synergism with or is complemented by other actions, thereby inducing a tangled pharmacological effect, giving the final picture of the puzzle-Figure 1 (e.g., when reading about the hypoglycemic action and LCF potential in reversing insulin resistance, its hypolipidemic, antiobesity or hepato-/vasoprotective effects must be taken into account and read, too, as they are congruent and convergent). Moreover, consulting the literature, it can be concluded that the existing in vitro/in vivo studies provide an important and motivating scaffold for future preclinical studies in order to elucidate the mechanism of action, as well as the introduction of this vegetal product in clinical trials.

## Data Availability

All the extracted data is presented in the manuscript.

## Abbreviations

ABCA1	adenosine triphosphate-binding cassette transporter 1
ABTS	2,2′-azino-bis(3-ethylbenzothiazoline-6-sulfonic acid)
AMPK	adenosine monophosphate-activated protein kinase
ARE	antioxidant response element
ATF3	activating transcription factor 3
C/EBPα	CCAAT/enhancer protein-α
C3G	cyanidin-3-o-glucoside
CAT	catalase
CI	confidence interval
COX-2	cyclooxygenase-2
CVDs	cardiovascular diseases/disorders
DM	diabetes mellitus
DNA	deoxyribonucleic acid
DPPH	2,2-diphenyl-1-picrylhydrazyl
DW	dry weight
FoXO1	forkhead box protein O1
FRAP	ferric reducing ability of plasma
FW	fresh weight
GAE	gallic acid equivalent
Gclc	glutamate-cysteine ligase catalytic subunit
GPx	glutathione peroxidase
HbA1c	glycated hemoglobin
HDL-C	high-density lipoprotein cholesterol
HFD	high fat diet
HO-1	heme oxygenase-1
HPLC	high-performance liquid chromatography
HR	heart rate
IC_50_	half maximal inhibitory concentration
IL	interleukin
iNOS	inducible nitric oxide synthase
IRS-1	insulin receptor substrate
LC	*Lonicera caerulea* L.
LCF	*Lonicera caerulea* L. fruits
LDL-C	low-density lipoprotein cholesterol
LPS	lipopolysaccharides
MCP-1	monocyte chemoattractant protein-1
MIP-1α	macrophage inflammatory protein
miR-33	microRNA 33
Mn-SOD	manganese-dependent SOD
mRNA	micro-ribonucleic acid
MS/MS	tandem mass spectrometry
NAD+	nicotinamide adenine dinucleotide
NADPH	nicotinamide adenine dinucleotide phosphate
NAFLD	non-alcoholic fatty liver disease
NASH	nonalcoholic steatohepatitis
NfκB	nuclear factor kappa-B
Nqo1	NAD(P)H dehydrogenase [quinone] 1
Nrf2	nuclear factor E2-related factor 2
oxHLIA	oxidative hemolysis inhibition
PDA	photodiode array
PGE2	prostaglandin E2
PI3K	phosphoinositide 3-kinase
PPAR-γ	peroxisome proliferator-activated receptor γ
ROS	reactive oxygen species
SFJP	sugar-free juice products
SMD	standardized mean difference
SOD	superoxide dismutase
SREBP2	sterol regulatory element-binding protein 2
T2DM	type 2 diabetes
TBARS	thiobarbituric acid reactive substances
TC	total cholesterol
TE	Trolox equivalent
TG	triglycerides
TNF-α	tumor necrosis factor-α
UPCL	ultra-performance liquid chromatography
VO_2_	oxygen consumption
